# Potential Pre-Leukemic Mutations in *PPM1D* Confer Chemotherapy Resistance to Aged HSC Clones

**DOI:** 10.1097/HS9.0000000000000171

**Published:** 2019-01-07

**Authors:** Michael D. Milsom

**Affiliations:** 1 Division of Experimental Hematology, German Cancer Research Center (DKFZ), Heidelberg, Germany.; 2 Heidelberg Institute for Stem Cell Technology and Experimental Medicine (HI-STEM), Heidelberg, Germany.

There has been a recent explosion in interest in the topic of non-malignant clonal hematopoiesis (CH), which arises in apparently healthy humans during the process of aging, and is frequently referred to in the literature as clonal hematopoiesis of indeterminate potential (CHIP) or age-related clonal hematopoiesis (ARCH).^1^ The growing activity in this research field has predominantly been driven by the observation that the incidence of CH correlates with risk of developing diseases such as atherosclerosis, stroke and hematologic malignancies, suggesting that a causal relationship may exist. However, for the vast majority of the somatic mutations that are frequently found in dominant age-associated clones, it is unclear why the mutant clone enjoys a selective growth advantage over its normal counterparts. Two recent studies, from the groups of Benjamin Ebert and Margaret Goodell, independently studied the biological relevance of mutations within exon 6 of the gene encoding protein phosphatase Mg2+/Mn2+ 1D (*PPM1D*), since analogous mutations are frequently found in CH.^2,3^

*PPM1D*, which is also known as wild-type p53-induced phosphatase 1 (*WIP1*), had been previously identified as a potential oncogene in several solid tumor entities, including breast and ovarian cancer and neuroblastoma.^4^ The putative mechanism of action of *PPM1D* relates to its capacity to dephosphorylate regulatory residues of key proteins involved in mediating the DNA damage response (DDR), including p53. Thus, aberrant activation of *PPM1D* would result in down-modulation of the DDR, potentially rendering cells resistant to various forms of DNA damage. In line with this hypothesis, both groups found that mutations in *PPM1D* were enriched in CH where the individual had prior exposure to chemotherapy, for example in the treatment of solid cancer or lymphoid malignancies. These mutations were also found in samples from patients with therapy-related MDS and secondary AML, suggesting that *PPM1D* mutant clones can act as the cell of origin for hematologic malignancies arising as a consequence of chemotherapeutic challenge.

Using gene editing to recapitulate truncating mutations in exon 6 of *PPM1D* in AML cell lines, both groups could observe that basal levels of mutant *PPM1D* were elevated compared to the wild-type protein. Further experiments strongly supported the conclusion that a proteasome degradation signal was present at this site in the protein and that this was lost in mutant cells, resulting in increased stability of the protein. In line with this concept, an unbiased CRISPR/Cas9 screen for functional domains using tiled guides spanning the *PPM1D* gene, revealed that mutations in amino acids 400–585, corresponding to exon 6, conferred resistance to cytarabine treatment in an AML cell line. Cell lines harboring mutant *PPM1D* demonstrated increased viability and a selective growth advantage compared to their isogenic counterparts upon treatment with clinically relevant chemotherapeutic agents such as cytarabine, cisplatin, doxorubicin and etoposide. This corresponded to an attenuated DDR, as classical DNA repair intermediates such as phosphorylated γH2AX and TP53, were suppressed in mutant cells upon chemotherapeutic challenge. Phosphoproteome analysis revealed that truncated *PPM1D* resulted in differential phosphorylation of multiple DDR pathway members in response to DNA damage, including TP53, CHECK1, CHECK2 and MDM4. Both groups could show that this compromised DDR resulted in decreased levels of apoptosis in response to chemotherapeutic challenge, while the Ebert group could additionally characterize that the cell cycle arrest was curtailed in mutant cells following cytarabine treatment. Importantly, overexpression of wild-type PPM1D could also confer increased chemo-resistance to AML cell lines, strongly suggesting that the attenuated DDR in cells with mutant *PPM1D* is predominantly driven by increased levels of the enzyme, resulting from enhanced protein stability, as opposed to any alternative biological effect resulting from the mutation. Indeed, the Goodell group could also identify rare instances where both de novo and secondary AMLs were associated with copy number gains at the *PPM1D* locus, as opposed to mutations in *PPM1D*.

Both groups also employed in vivo mouse models to demonstrate that the PPM1D could mediate a selective growth advantage following chemotherapy, and that this was therefore the likely basis for the clonal expansion of mutant cells. The Ebert group used CRISPR/Cas9 to induce an exon 6 mutation in primary c-Kit^+^ bone marrow progenitor cells. Following co-transplantation of these gene edited progenitors with wild-type competitor cells, and subsequent reconstitution of the recipient animal, cytarabine treatment resulted in the in vivo expansion of mutant cells at the expense of their normal counterparts. Likewise, the Goodell group generated a *Ppm1d* mouse model with a truncating mutation at R451, analogous to mutations commonly found in CH. The heterozygous R451 mutation did not compromise hematopoiesis in any discernable way. However, mutant cells again were able to outgrow their wild-type counterparts in a competitive transplant setting, but only when recipient mice were challenged with cisplatin. Interestingly, Ppm1d R451 mutant stem cells did not demonstrate an enhanced growth advantage in response to proliferative stress, as assessed by performing serial transplantation assays. In fact, Ppm1d cells demonstrated a mild engraftment defect compared to wild-type cells. This contrasts with the impact of loss of function of the DNA methyltransferase, Dnmt3a, another commonly mutated gene in CH, that results in enhanced stem cell engraftment and enhanced self-renewal. Thus, the mechanisms via which CH can be established are highly context-dependent and are driven by the nature of the altered biology conferred by CH-associated mutations. Importantly, both groups could also show that the chemo-resistance conferred by *PPM1D* mutations could be overcome by the use of GSK2830371, an allosteric inhibitor of PPM1D activity. This opens the possibility that PPM1D mutant AML or MDS cells could be sensitized to chemotherapy via such an approach, although it is not clear whether this will result in an effective therapeutic window, since wild-type cells may also be subject to increased toxicity in response to such a regimen.

**Figure 1:**
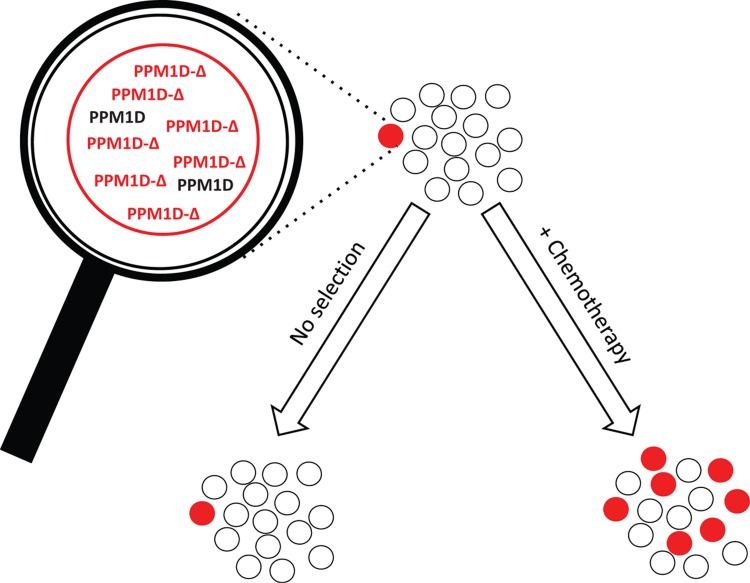
Mutations in exon 6 of *PPM1D* result in elevated levels of the protein due to increased protein stability, and a resulting increased resistance to DNA-damaging agents. Treatment with chemotherapy subsequently results in the selective expansion of *PPM1D* mutant clones.

Overall, this phenomenon is somewhat reminiscent of cases of CH that are driven by mutations in TP53, which are also enriched in individuals that have been exposed to chemotherapy. However, TP53 mutations are also frequently associated with large karyotypic abnormalities, which are not seen in *PPM1D* mutant AML/MDS. Therefore, while TP53 and PPM1D mutations likely have some overlapping features in the mechanism via which they promote chemo-resistance, the observed differences in genome stability suggest that there are also important divergent functions resulting from these mutations. This may relate to the nature of the precise DDR components that are impacted upon by p53 and PPM1D mutations, or may relate to the reported impact of PPM1D on p38MAP Kinase signaling, which can also profoundly impact on cell survival. A more detailed analysis of the mutation profile of PPM1D stem and progenitor cells following chemotherapeutic challenge is probably warranted in order to establish whether such mutations will enhance the survival of cells that harbor a high mutation burden. Such an analysis would clearly shed light on whether mutant PPM1D is frequently observed in therapy-induced AML/MDS because it facilitates acquisition of cooperating mutagenic hits, or whether it is simply a benign passenger during the transformation process Figure 1.
